# Patient and care partner perspectives and preferences related to myasthenia gravis treatment: A qualitative study

**DOI:** 10.1002/hsr2.70081

**Published:** 2024-09-24

**Authors:** Margaret Yung, Pushpa Narayanaswami, Jacqueline Pesa, Zia Choudhry, Louis Jackson, Kathleen L. Deering, Jamie Sebaaly, Jordan Richardson, Josh Feldman, Wesley Peters, Melina Taylor, Allison Foss, Bruce West, Lisa Shea, Gabrielle Geonnotti, Raghav Govindarajan

**Affiliations:** ^1^ EPI‐Q, Inc. Chicago Illinois USA; ^2^ Beth Israel Deaconess Medical Center/Harvard Medical School Boston Massachusetts USA; ^3^ Janssen Scientific Affairs, LLC Titusville New Jersey USA; ^4^ Inspire Insights Arlington Virginia USA; ^5^ CorEvitas, Part of Thermo Fisher Scientific Waltham Massachusetts USA; ^6^ Myasthenia Gravis Association Kansas City Missouri USA; ^7^ Patient Engagement Research Council (PERC) Member USA; ^8^ HSHS Medical Group Multispecialty Care O'Fallon Illinois USA

**Keywords:** myasthenia gravis, patient preference, patient perspective, symptoms, treatment

## Abstract

**Background and Aims:**

Due to the high symptom and treatment burden in myasthenia gravis (MG), understanding patient and care partner perspectives and preferences is crucial.

**Methods:**

This study used voice analysis and virtual focus groups to understand patient and care partner experiences with MG‐related symptoms, treatments, and preferences. The voice analysis via social media listening used artificial intelligence‐powered tools to gather and structure public digital conversations on MG. Focus groups included people living with MG and care partners who completed a questionnaire and participated in a 1‐h virtual session facilitated using a semi‐structured interview guide. Qualitative data were aggregated, transcribed, and thematically analyzed.

**Results:**

The voice analysis examined 11,554 posts from 8321 individuals, discussing MG symptoms, treatments, and burden. Of 7563 symptom‐related posts, 5902 (78%) conveyed negative, 1427 (19%) neutral, and 234 (3%) positive sentiment. The most frequently mentioned symptoms were categorized as dysarthria, muscle weakness, and dysphagia. MG treatment sentiment analysis identified 6667 posts (67%) as neutral, 2887 (29%) as negative, and 350 (4%) as positive. For the focus groups, 15 individuals (12 patients and 3 care partners) completed the questionnaire and 14 participated in the virtual focus group sessions. The 15 participants who completed the questionnaire prioritized treatment convenience, symptom control for improved quality of life, and preventing potential MG crises in their current treatment. New treatment expectations included increased effectiveness, less frequent dosing, faster onset, and fewer side effects. Participants were also receptive to wearable medication delivery systems placed on the body and valued direct involvement in treatment decisions.

**Conclusion:**

Patients and care partners are often negatively impacted by MG symptoms and value convenient and fast‐acting treatments that control symptoms with minimal side effects. Considering patient preferences may help optimize treatment decisions and improve patients' overall well‐being and satisfaction in their care.

## Key points

- Recognizing and valuing patients' expertise in their health and treatment preferences is vital, especially since factors such as daily functioning, symptoms, and treatment administration impact patients' daily lives and influence myasthenia gravis (MG) treatment management.
- Patients and care partners are often negatively impacted by MG symptoms and prioritize convenient treatments that control symptoms with minimal side effects.
- A greater understanding of these preferences and perspectives may encourage more personalized treatment plans and improved patient experiences in MG.

## 1 INTRODUCTION

Myasthenia gravis (MG) is a rare, chronic, autoimmune neuromuscular disorder affecting approximately 14–36 people per 100,000.^1^, ^2^ MG can occur at any age, impacting various aspects of a patient's life such as daily activities (e.g., brushing teeth and eating), vision, and physical abilities.^3^ While most MG manifests as weakness of the extraocular muscles (ocular MG), approximately 85% will present with or progress to generalized MG that affects the face, neck, and/or extremities.^4^, ^5^ In more severe cases, MG crises can develop, leading to weakness in respiratory muscles, resulting in hospitalization and a need for ventilatory support.^6^

The symptoms of MG are often variable and unpredictable, causing patients to be frustrated and desire greater control over their condition.^7^ Treatment typically includes symptomatic cholinesterase inhibitors, immunosuppressive therapies, and other therapies such as rituximab, intravenous immunoglobulin, complement C5 inhibitors, fragment crystallizable receptor inhibitors, therapeutic plasma exchange, and thymectomy.^8^ These treatment options have differences in sustained response, tolerability, routes of administration, administration schedule, and cost. However, evidence suggests that many patients still experience disease‐related impairments, impacting their mobility, mental well‐being, and overall quality of life.^2^, ^9^, ^10^ Fluctuating, fatigable muscle weakness has a particularly debilitating impact on patients with MG and can continue to affect those who are undergoing treatment.^11^

Due to the heterogeneity of MG, no single treatment approach is best for all patients. Each patient has a unique lived experience, and their perspective of their disease can evolve over time. Some patients with MG report feeling disconnected with their healthcare providers, contributing to the belief that their healthcare providers do not understand what is important to them.^2^ Recognizing patients as experts in their own health, treatment preferences, and health states is important in the treatment decision‐making process, especially in rare diseases. Various aspects, such as daily function, symptoms, and treatment administration, can influence a patient's perspective, and considering these factors may lead to improved quality of life and overall patient satisfaction in patients with MG.^12^, ^13^ Additionally, although information on MG care partner perspectives is lacking, patients with MG rely on care partners four times more than the general population.^14^ Understanding the care partner's viewpoint is important, and can provide a holistic perspective on optimizing treatment decisions for patients with MG.

Existing literature has explored patient‐reported MG disease burden using traditional research methods including surveys and structured patient‐reported outcome measures.^15^, ^16^, ^17^ However, qualitative research on patients' lived experiences and perspectives remains limited. Few studies explore the patient perspective on MG symptom burden and treatment goals.^2^, ^11^ This gap in the literature underscores the need for more comprehensive studies that explore more subjective aspects of patients living with MG. Additionally, in an increasingly connected world, patients often share their experiences on open social networks (e.g., Twitter and Instagram) and online communities.^18^ To date, the use of sentiment analysis or social media listening to capture patient perspectives in MG or other neurological diseases is not well‐ documented. Using this approach may allow researchers a valuable opportunity to gather data on patient experiences and priorities that traditional research methods may overlook. Expanding qualitative research in MG can enhance the understanding of the MG patient experience and enable healthcare providers to deliver more empathetic, patient‐centered care.

This study aims to understand patients' emotional perspectives around treatment and symptom burden in MG. It also explores patient and care partner treatment preferences, concerns, and attributes for current and new MG treatments, including valued aspects of emerging treatments in MG.

## 2 METHODS

This study comprised a two‐part methodology. The first part included a patient and care partner voice analysis via social listening to understand their sentiment, perspectives, and experiences with MG‐related symptoms and treatment burden. This informed the conceptualization and content development of the second part of the study, which employed virtual focus groups to understand patient and care partner preferences, considerations, and expectations around current and new treatments in MG. Participants in the focus groups provided their informed consent through a consent and release form, which assured the confidentiality of their information in line with Health Insurance Portability and Accountability Act regulations. Additionally, since all collected data from the focus groups and real‐world voice analysis were anonymized, there was no requirement for an institutional review board. This study was sponsored by Janssen Pharmaceuticals.

### 2.1 Real‐world voice analysis

A targeted social media analysis utilized advanced search, data extraction, and artificial intelligence (AI)‐powered tools to harvest, mine, and analyze online discussions about MG among adults in the United States from August 2020 to August 2022. Data were collected from key Inspire.com communities, including an MG‐specific one, and open social media such as Reddit, Instagram, X (formerly known as Twitter), and Facebook. Open social media only included publicly accessible pages from these platforms. For example, private Facebook pages requiring approval to join the group were not included in the data collection. Inspire has facilitated research on various health communities and is a leading social health network, in which patients, families, and care partners can connect with one another for support and practical information.^19^, ^20^, ^21^, ^22^, ^23^, ^24^ The study examined online discussions related to both current and past treatment and symptom experiences, and also explored the overall patient journey, emotions, and behaviors within the context of MG. Inclusion criteria were posts from individuals with MG or their care partners discussing their experiences with MG. Care partners were identified using search terms representing a close friend or relative. Terms such as “significant other,” “partner,” and “friend” were tagged using AI and then manually reviewed for relevancy and context related to identification as a care partner for MG. Online discussions were limited to those available publicly and originating from the United States. Any posts from healthcare professionals, news sites, and researchers were excluded. Diagnoses of MG and medical information were not verified in these posts. Data were collected using predefined key listening terms, Boolean strings related to MG, standard‐of‐care treatments, and key MG burden terms (see Supporting Information: 1).

**Figure 1:**
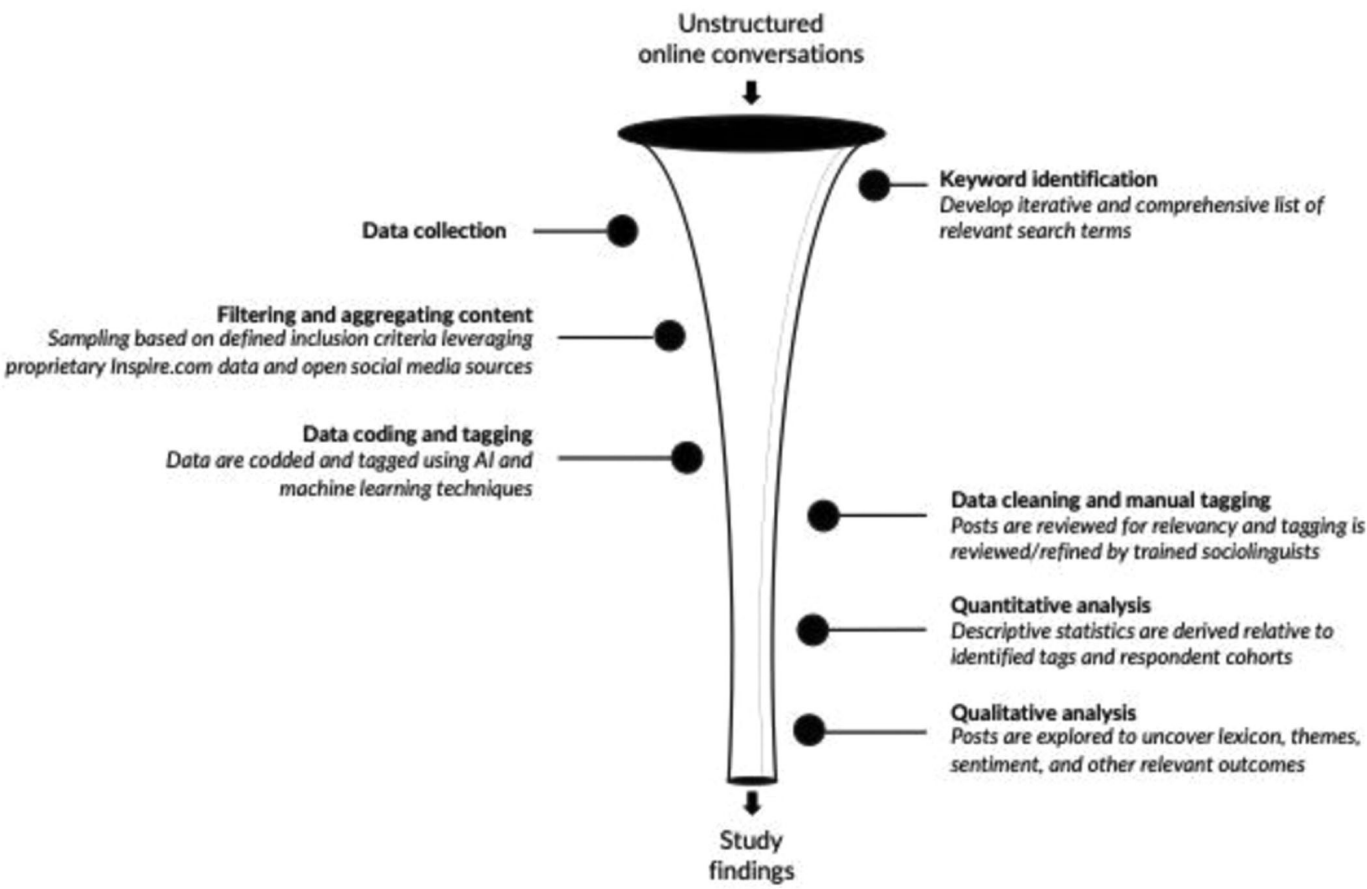
Overall framework for the real‐world voice analysis.

Brandwatch Analytics, a social media intelligence tool that leverages proprietary AI to extract and analyze data from various social media platforms, was used in this study.^25^ Machine learning and AI were employed to tag, segment, and categorize data based on topic. Additionally, natural language processing was utilized for sentiment analysis, characterizing posts as positive (expressing satisfaction, joy, or other positive emotions related to MG experiences such as positive responses to treatments, successful management of symptoms, or supportive interactions within the community), negative (conveying dissatisfaction, frustration, or other negative emotions such as challenges in treatment, difficulties in symptom management, or emotional strain experienced by patients or care partners), or neutral (posts that express neither positive nor negative sentiments such as informational or factual posts without a distinct emotional tone, providing updates on the MG journey, treatment plans, or general discussions without emotional connotations). Symptom‐related negative sentiments were then categorized into five emotion‐based groups (anger, fear, disgust, surprise, and sadness) that were identified by AI at the start of the sentiment analysis process. Following this automated process, manual accuracy checks of each post were conducted to ensure additional recognition of nuanced linguistic patterns and enhance the reliability of the sentiment analysis results. Qualitative analysis was conducted using trained sociolinguists in combination with advanced natural language processing to assess context, frequency, and sentiment surrounding key topics and themes occurring in online conversations. Figure 1 outlines the overall framework for the real‐world voice analysis.

### 2.2 Focus groups

Three virtual focus groups, comprising members from the Patient Engagement Research Council (PERC) program, met in June 2023. The PERC program, led by Janssen Pharmaceuticals, engages a diverse group of patients with specific health conditions, as well as care partners of such patients. Their collective knowledge provides insights to inform research. PERC members were screened for inclusion using an MG‐specific online screener followed by a subsequent telephone interview. Participant eligibility for these focus groups included being at least 18 years old, living in the United States, and having a self‐reported diagnosis of MG. Care partners were required to provide caregiving to a patient meeting the same eligibility criteria. PERC members were also recruited to ensure diversity in age, gender, race/ethnicity, time since MG diagnosis, disease severity, educational background, and treatment experience.^26^ A semi‐structured discussion guide, informed by a targeted literature review and input from a steering committee of clinical experts and a patient advocate (who is also a person with MG), was developed and used to elicit open and honest participant feedback and opinions about treatment preferences related to MG. Participants were also asked to complete an eight‐item pre‐focus group questionnaire centered around MG treatment choices (e.g., attributes, concerns, goals, and expectations) before the focus group sessions (see Supporting Information: 2). Questions consisted of ranking exercises, Likert scale questions, multiple choice, and a question from a previously validated scale, known as the Control Preferences Scale,^27^ that assessed participants' preferred role in treatment decisions. For the ranking exercises, participants ranked treatment attributes in order of importance from the most important attribute (1) to the least important attribute (10); treatment goals were ranked from 1 (most important) to 9 (least important). From a list of statements, participants were also asked to select the five most concerning factors about their current or most recent treatment, in addition to the five key expectations for a new or emerging MG treatment. Finally, Likert scale questions, based on a 5‐point scale, asked participants about their preferred options for new or emerging treatments (1, most preferred; 5, least preferred), along with factors most important to them in deciding the next possible MG treatment (1, not at all important; 5, very important). Participant responses to the pre‐focus group questionnaire were used to prompt for additional input during the sessions. Each virtual focus group session lasted 2 h and was conducted by CorEvitas, a part of Thermo Fisher Scientific, a science‐led, real‐world data intelligence and patient experience insights company.

The results of the pre‐focus group questionnaire used aggregated descriptive statistics to report participant demographics and responses to the questions, no statistical tests were conducted. For ranking exercises, respondent answers were averaged for each treatment attribute/goal and then sorted by the lowest to highest averaged value. The lower the averaged value, the more important or preferred the treatment attribute or factor was to respondents. Qualitative data from the focus groups were recorded, transcribed, and analyzed by experienced CorEvitas research specialists using conversational (narrative) analysis and by direct observation, both during data collection and through transcript analysis to understand patient and care partner preferences in MG. All transcripts were coded based on concepts using a qualitative research analysis program (MAXQDA).

## 3 RESULTS

### 3.1 Real‐world voice analysis

**Figure 2:**
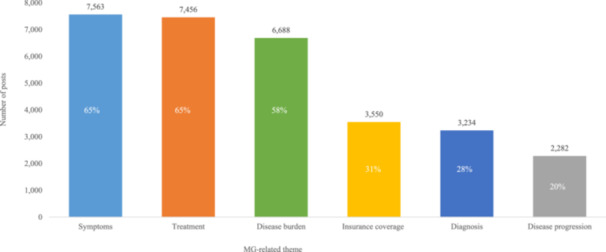
Top MG‐related discussion themes identified in the voice analysis out of a total of 11,554 distinct posts. Note that patients may have discussed more than one theme. MG, myasthenia gravis.

In total, 11,554 distinct posts were identified from 8321 unique contributors who met the inclusion criteria. The primary discussion themes included MG symptoms (7563 posts [65%]) and treatments (7456 posts [65%]), and disease burden (6688 posts [58%]) (Figure 2). Patients may have discussed more than one theme. Patients also used open‐social platforms such as Reddit, Instagram, and X (formerly known as Twitter) to discuss MG‐related topics, including insurance coverage of MG (3550 posts [31%]), MG diagnosis (3234 posts [28%]), and disease progression (2282 posts [20%]).

#### 3.1.1 Symptom‐related posts

**Figure 3:**
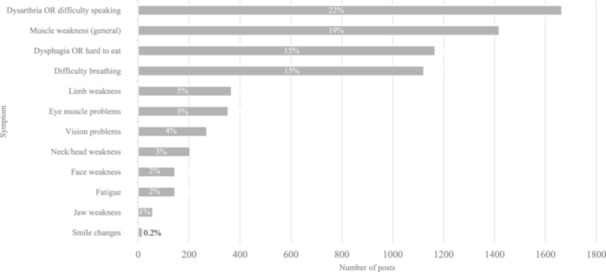
Symptom burden. MG symptoms were identified and categorized from the voice analysis. Note that 629 posts are not shown in this figure as they were symptom‐related posts without reference to specific symptoms. MG, myasthenia gravis.

**Figure 4:**
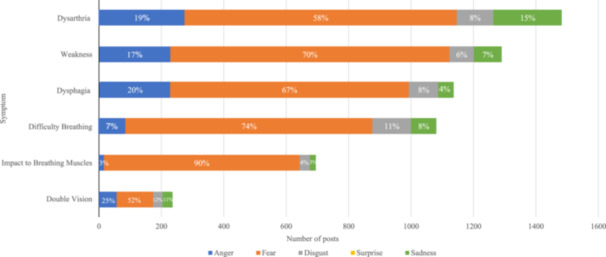
Negative symptom sentiment drivers. Symptom‐related negative sentiments were categorized into five emotion‐based groups: anger, fear, disgust, surprise, and sadness. Note that posts may be characterized as having multiple combinations of sentiments and the percentages in each category may not sum to 100% due to rounding.

Patients used everyday language to describe symptoms, and of the 7563 posts about MG symptoms (65%), the most frequently mentioned ones were categorized as dysarthria or difficulty speaking (1665 posts [22%]), muscle weakness (1419 posts [19%]), dysphagia or hard to eat (1167 posts [15%]), and difficulty breathing (1123 posts [15%]) (Figure 3). Among these symptom‐related posts, a majority expressed negative sentiments about MG symptoms (5902 posts [78%]), followed by posts that expressed neutral sentiment (1427 posts [19%]). Only 234 posts (3%) expressed positive sentiment. Posts with neutral sentiment often shared information about MG symptoms (685 posts [48%]), while posts discussing symptom relief (143 posts [61%]) were considered in the positive sentiment category. The five emotion‐based groups as identified by AI for symptom‐related negative sentiments included anger, fear, disgust, surprise, and sadness (Figure 4). The most frequent negative sentiment expressed for the symptom‐related posts was fear (4065 posts [69%]). Out of the posts for each symptom, muscle weakness had the highest number of posts expressing fear (900/1289 posts [70%]), followed by dysarthria (863/1478 posts [58%]) and difficulty breathing (796/1079 posts [74%]).

Other aspects of the patient's MG‐related symptom experience included 2089 posts (28%) discussing cognitive or emotional burden due to unresolved MG symptoms, in addition to 353 posts (5%) discussing fluctuations in symptoms. Patients also described the impact of MG symptoms on their quality of life, covering issues such as fatigue and work limitations (382 posts [5%]), challenges in pursuing hobbies due to muscle weakness (416 posts [6%]), and missed time with loved ones due to the lack of diagnosis and inadequate symptom control (347 posts [5%]). Furthermore, in 205 posts (3%), patients on open social platforms expressed concerns about their symptoms being dismissed by their clinicians.

#### 3.1.2 Treatment‐related posts

**Figure 5:**
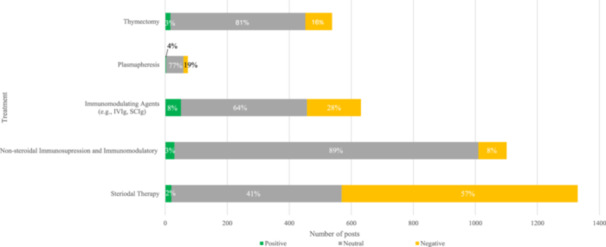
Treatment sentiment analysis. Treatments were categorized as positive, neutral, or negative sentiments. Search terms utilized encompassed variations in names and drug classes for each category in the figure. IVIg, intravenous immunoglobulin; SCIg, subcutaneous immunoglobulin.

Of the 9904 treatment‐related posts, sentiment analyses identified 6667 posts (67%) as neutral sentiment, 2887 posts (29%) as negative sentiment, and 350 posts (4%) as positive sentiment. The most common negative sentiments were related to adverse events (771 posts [26%]), unstable disease control (573 posts [19%]), and treatment inertia (235 posts [8%]). For treatment inertia, patients expressed frustration around the lack of treatment change, cycling through treatments, or the persistent use of the same treatment type regardless of treatment effectiveness. Treatment‐related posts that expressed positive sentiment often discussed relief and excitement about the efficaciousness of MG treatments. Sentiment analysis for standard‐of‐care MG treatments (Figure 5) indicated substantial negative sentiment related to corticosteroid therapy, with 761 negative sentiment posts (57%) compared with 20 that had positive sentiment (2%). The reasons for negative sentiments included patients expressing fear or discussing their concerns about experiencing side effects such as “moon face” and weight gain due to corticosteroid use.

### 3.2 Focus groups

Fifteen PERC members (12 patients and 3 care partners) completed the eight‐item pre‐focus group questionnaire and 14 participated in the virtual focus group sessions. The mean age was 52 years (standard deviation 12.4) for patients and 63 years (standard deviation 6.4) for care partners. Nine participants (60%) were female, nine (60%) were White, and nine (60%) held a bachelor's degree or higher. Eight respondents (5 patients and 3 care partners) reported that they or their loved one had received an MG diagnosis between 1 and 5 years previously. Demographics for the participants completing the pre‐focus group questionnaire are presented in Table 1.

**Table 1:** Demographics of focus group participants.^a^

	Total patients (*N* = 12)	Total care partners (*N* = 3)
Age, mean (SD), years	52 (12.4)	63 (6.4)
Gender, *n* (%)		
Female	6 (50)	3 (100)
Male	5 (42)	0
Nonbinary	1 (8)	0
Race/ethnicity, *n* (%)		
White	6 (50)	3 (100)
Black/African American	4 (33)	0
Other	2 (17)	0
Education level, *n* (%)		
High school	2 (17)	1 (33)
Some college/trade school	3 (25)	0
Bachelor's degree	5 (42)	1 (33)
Post‐graduate	2 (17)	1 (33)
Time since diagnosis		
<1 year ago	0	0
1–5 years ago	5	3^b^
6–10 years ago	3	0
11–15 years ago	0	0
16–20 years ago	0	0
≥21 years ago	4	0

Abbreviations: MG, myasthenia gravis; SD, standard deviation.

^a^ Includes 15 participants who completed the pre‐focus group questionnaire.

^b^ Denotes care partner responses describing their loved one's MG.

#### 3.2.1 Pre‐focus group questionnaire

The three most important treatment attributes included avoiding or preventing an MG crisis, controlling MG symptoms, and reducing side effects from treatment (Table 2). The most concerning factors about their current or most recent treatment included managing MG symptoms (87%) and how well the treatment will work (87%), followed by potential exacerbations or MG crises (73%).

**Table 2:** Importance of treatment attributes.^a^

Ranking order	Treatment attribute	Mean score^b^
**1**	Avoiding or preventing an MG crisis	3.3
**2**	Controlling symptoms	3.8
**3**	Reducing side effects from treatment	4.7
**4**	Expected length of time the treatment will work	5.1
**5**	Cost of treatment	5.3
**6**	How long it takes before improvement starts	5.8
**7**	Impact on quality of life	6.3
**8**	Where the treatment is given	6.5
**9**	How often the treatment needs to be given	6.5
**10**	How the treatment is given	7.8

Abbreviation: MG, myasthenia gravis.

^a^ Includes 15 participants who completed the pre‐focus group questionnaire.

^b^ Lower scores indicate attributes that were more important to participant.

**Figure 6:**
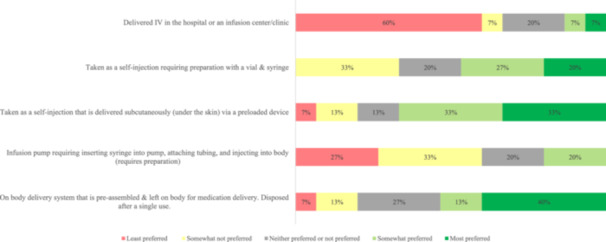
Preferred emerging MG treatment administration option. Question from pre‐focus group questionnaire: For a new or emerging myasthenia gravis treatment, how would you like it to be given to you/your loved one? Please rank each option in order from 1 (most preferred) to 5 (least preferred). [Likert scale 1–5, with pictures and explanations. Scale: 1 Most preferred, 2 Somewhat preferred, 3 Neither preferred or not preferred, 4 Somewhat not preferred, and 5 Least preferred]. Includes 15 participants who completed the pre‐focus group questionnaire. Note that percentages in each category may not sum to 100% due to rounding. IV, intravenous; MG, myasthenia gravis.

**Figure 7:**
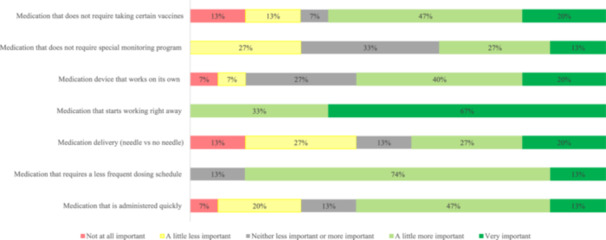
Important factors for next possible emerging MG treatment. Question from pre‐focus group questionnaire: How important are the following factors when making decisions about a next possible myasthenia gravis treatment for you/your loved one? [Likert scale 1–5. Scale: 1 Not at all important, 2A little less important, 3 neither less important or more important, 4A little more important, and 5 Very important]. Includes 15 participants who completed the pre‐focus group questionnaire. Note that percentages in each category may not sum to 100% due to rounding. MG, myasthenia gravis.

When asked about knowledge and expectations about new or emerging MG treatments, the most important expectations included reducing MG crises (87%) and having a medication that worked for a longer period of time (80%). For preferences on emerging treatment administration options, convenience was an important factor for participants, with 60% reporting that treatments administered intravenously were the least preferred option (Figure 6). For the most important factors about the next possible MG treatment, all participants somewhat preferred or mostly preferred a medication that worked rapidly and considered a less frequent dosing schedule as an important factor (Figure 7). When asked about shared decision‐making, 80% of participants preferred to share involvement with the treating physician or make the final selection after considering the physician opinion, 13% wanted to make the final treatment selection, and 7% wanted to leave all the treatment decisions to the physician.

#### 3.2.2 Insights from focus groups

##### Current MG treatments

In the pre‐focus group questionnaire, participants ranked treatment attributes based on the level of importance when deciding on MG treatment. Among the treatment attributes provided, quality of life ranked 7th compared with the other attributes (Table 2). However, during the focus group sessions, participants did not differentiate between quality of life and other treatment factors, such as minimizing side effects or preventing MG crises. Most participants believed that addressing any of the treatment attributes, as defined by the pre‐focus group questionnaire, would lead to an improvement in their quality of life. As an example, participants considered avoiding a crisis or hospitalization as important, but also believed that addressing other disease‐related aspects could prevent an MG crisis and improve overall quality of life. This included addressing both mental health impacts (e.g., depression, anxiety, and social isolation) and physical functional impacts (e.g., maintaining endurance, swallowing, speaking, and physical fatigue experienced during extended periods of sitting while undergoing treatment). Acceptance of dosing frequency varied, but participants stressed the significance of treatment convenience, and favored at‐home administration options over in‐clinic or hospital settings. Finally, aligned with the pre‐focus group questionnaire findings, managing symptoms was important as it could lead to an improvement in quality of life and prevent a potential MG crisis or flare‐up. Participants stated that symptom control impacted other aspects of MG including mental and physical fatigue, communicating concerns about addressing depression, anxiety, social isolation, and managing physical impacts such as mobility and functionality in tasks like swallowing and speaking.

##### New treatments

When asked about expectations in new MG treatments, participants valued symptom control and achieving disease stability in their day‐to‐day lives. While dosing frequency was an important factor in the pre‐focus group questionnaire, participants indicated their willingness to accept more frequent dosing if it resulted in fewer symptom fluctuations, reduced side effects, or fewer crises/flare‐ups. Additionally, participants expressed a greater willingness to have more frequent dosing if the treatment could be administered at home. The acceptability of treatment effect onset times varied (e.g., 2 months vs. 9 months) and participants felt it was helpful to be informed about these onset times before treatment initiation. Overall, participants desired a faster treatment effect onset time, especially when experiencing an MG crisis.

Participants were presented with five administration options in the pre‐focus group questionnaire, and their choices were further examined during the focus group sessions. These administration options focused on injectable drug delivery systems and included self‐injections via autoinjector, self‐injections via vial and syringe, infusion pump, intravenous delivery at a hospital or clinic, and an on‐body delivery system (OBDS). During the focus groups, individuals who prioritized the autoinjector expressed value in its practicality during travel and ease of use, but some participants who disliked this option had concerns about its safety, the self‐administration process, and how to navigate side effects such as injection‐site reactions. For OBDS, all participants stated that they had no prior experience with this delivery system, but several were familiar with the technology. In general, participants were receptive to its ease of use and convenience to fit their lifestyles. Concerns about the OBDS included duration of wearing the device, potential skin reactions due to the adhesive, and proper device disposal (e.g., waste product or recycled). Participants expressed interest in understanding usage frequency, treatment efficacy, and associated side effects. Finally, when asked about self‐injections via vial and syringe in the focus groups, participants with prior experience using this delivery method expressed greater willingness to try this delivery method. Concerns included preparation challenges (e.g., accurate measurements and dexterity limitations), maintaining temperature stability, and a higher risk of human error. Those who stated they feared needles expressed unwillingness to use self‐injections as a mode of administration.

##### Shared decision‐making

Participants reiterated their pre‐focus group questionnaire responses about wanting to be highly involved in treatment decisions. Most preferred to either actively participate in treatment decisions alongside their physician or opted to make the final decision themselves, after seriously considering their physician's recommendations. Several participants also mentioned the importance of consulting partners, family members, other care partners, and other care team members in their treatment decisions.

## 4 DISCUSSION

This study used a two‐pronged approach to understand and provide a unique portrayal of the MG symptom experience, treatment burden, and treatment preferences among patients and care partners.

Patient sentiment toward MG symptoms was generally negative, often associated with feelings of fear. This sentiment may be linked to unresolved MG symptoms and the adverse impact symptom fluctuations can have on patients' physical well‐being and overall quality of life. These findings align with previous research that explored patient perspectives on MG symptom burden, highlighting the substantial impact that fluctuating and unpredictable symptoms have on patients' daily lives.^2^, ^11^ In the study by Law et al, patients stated that they felt that they were in a constant state of adaptation and were required to continually make compromises, such as needing to cancel arrangements with others due to a potential flare.^11^ The fluctuating nature of MG has also been found to affect other aspects of patient's daily lives and cause challenges in navigating through social limitations, feelings of depression, anxiety, and guilt.^2^, ^28^, ^29^

The unpredictability of these symptoms associated with MG has also been a challenge for clinicians who care for these patients. Although treatment guidelines have been established for MG, there remains no consensus on the definition of optimal disease control.^8^, ^11^, ^30^ Patients and clinicians may hesitate to change treatments because of concerns over adverse events and uncertainty, resulting in suboptimal care.^11^ However, in our study, certain posts indicated patients' frustrations due to the lack of treatment change or the persistent use of the same type of treatment (i.e., treatment inertia). Some patients also expressed feeling their symptoms were disregarded by their clinician. This may suggest a clinician–patient disconnect, emphasizing the need for treatment discussions that factor in considerations about the patients' quality of life and their treatment priorities.

There is also limited research in MG on patient treatment preferences and the impact of a patient's quality of life on their treatment decisions.^28^, ^31^, ^32^ In our focus groups, the relationship between treatment factors and possible improvement in quality of life was a driving factor for our participants' treatment decisions. Participants who completed the questionnaire believed that addressing other factors would enhance their quality of life. For instance, improvements in symptom control, treatment effectiveness, and fewer side effects could help enable them to resume former activities. Findings from this study also emphasized the importance of treating holistically and factoring in patient preferences that could result in improved quality of life.

Participants in our focus groups identified treatment efficacy and convenience as the most important treatment factors for potential new therapies. However, participants also reported they would consider bearing greater treatment burden, such as more frequent dosing, if it resulted in greater symptom control, fewer side effects, or scheduling flexibility. There remains continual uncertainty in MG management, and patients often make trade‐offs that impact their day‐to‐day decisions.^11^ Being able to provide patients with a greater sense of control through more convenient and effective treatment options is needed to address the ongoing challenges faced by patients with MG.

Incorporating individual patient preferences into their treatment plans has been shown to have numerous benefits, including enhancing patient satisfaction and improving health outcomes.^33^, ^34^ With increasing emerging MG treatments that could potentially offer better safety profiles, efficacy, and a variety of administration modalities compared to traditional therapies, it is critical to understand patient treatment preferences. Therefore, a more extensive study, such as a discrete‐choice experiment, would allow the opportunity to further explore trade‐offs patients would make in the treatment decision process, and offer deeper insights into patient preferences in MG and specific treatment attributes influencing their decisions.

### 4.1 Limitations

For the social media analysis, the population may underrepresent those not engaged in social media, or those who do not have access to the internet or electronic devices, resulting in response bias. Other limitations included the inability to verify diagnosis, severity, or type of MG, and lack of sociodemographic data for the data collected through open social platforms. Additionally, Brandwatch may not capture every mention of MG and is limited to the data query designed for this study. The data gathered by Brandwatch may not be representative of all online conversations about MG or be indicative of similar populations at different points in time. For the focus groups, participants were part of the sponsor's PERC and consisted of individuals who self‐identified as having MG. These members were open to participating in research, potentially resulting in bias during the discussions. Furthermore, the inclusion of care partner input in this study was limited to participants who were part of the sponsor's PERC. The sample size was small, making it difficult to differentiate among the responses. The results should be viewed as hypothesis‐generating rather than hypothesis‐confirming. Finally, as this was a US‐based study, the topics and results presented may not be generalizable outside US populations.

## 5 CONCLUSIONS

This research highlights the importance of the patient perspective, understanding symptom and treatment burdens, and patient and care partner treatment preferences in MG. Patient sentiment toward MG symptoms was generally negative. When asked about important factors in deciding MG treatment, patients prioritized symptom control, greater treatment flexibility, and more convenient modes of administration. They recognized that while these factors were important, they were hopeful that addressing these factors would lead to an improved quality of life. These findings encourage clinicians not just to consider managing MG symptoms, but to treat holistically, and to better understand how treatment decisions will impact patients' overall quality of life. Full consideration of patient preferences may help better navigate the unpredictability of MG, and is an opportunity to improve patients' overall well‐being, enhance patient satisfaction, and optimize treatment decisions.

## AUTHOR CONTRIBUTIONS

**Margaret Yung**: Conceptualization; investigation; methodology; project administration; writing—original draft. **Pushpa Narayanaswami**: Methodology; writing—review and editing. **Jacqueline Pesa**: Conceptualization; funding acquisition; methodology; supervision; writing—review and editing. **Zia Choudhry**: Conceptualization; methodology; funding acquisition; writing—review and editing; supervision. **Louis Jackson**: Methodology; writing—review and editing. **Kathleen L Deering**: Conceptualization; investigation; methodology; project administration; supervision; writing—review and editing. **Jamie Sebaaly**: Methodology; writing—review and editing. **Jordan Richardson**: Data curation; formal analysis; writing—review and editing. **Josh Feldman**: Project administration; writing—review and editing. **Wesley Peters**: Project administration; writing—review and editing. **Melina Taylor**: Data curation; formal analysis; writing—review and editing. **Allison Foss**: Methodology; writing—review and editing. **Bruce West**: Methodology; writing—review and editing. **Lisa Shea**: Methodology; writing—review and editing. **Gabrielle Geonnotti**: Methodology; writing—review and editing. **Raghav Govindarajan**: Methodology; writing—review and editing.

## CONFLICT OF INTEREST STATEMENT

GG, JP, LJ, LS, and ZC are current employees of Janssen Scientific Affairs, LLC. KLD, MY, and JS are employees of EPI‐Q, Inc., which received funding from Janssen Scientific Affairs, LLC, associated with the development and execution of this study. JF and JR are current or former employees of Inspire, which received funding associated with the development and execution of this study. MT and WP are employees of CorEvitas, which received funding from Janssen Scientific Affairs, LLC, associated with the execution of this study. PN has served consultancy/advisory roles for Argenx, Alexion, Dianthus, GSK, ImmunAbs, Janssen, Novartis, UCB; received research funding from Alexion, Dianthus, Janssen, Patient Centered Outcomes Research Institute (PCORI), UCB; and served on the data safety monitoring board for Sanofi. AF has received honorarium for advisory boards from Argenx, Immunovant and UCB. BW is a Patient Engagement Research Council (PERC) member and has received payment from Janssen Scientific Affairs, LLC. RG has received honoraria for advisory boards from Alexion, Argenx, UCB, Janssen, Alexion and speaking‐related honoraria from Alexion, Argenx, and UCB.

## ETHICS STATEMENT

Participants in the focus groups provided their informed consent through a consent and release form, which assured the confidentiality of their information in line with Health Insurance Portability and Accountability Act regulations. Additionally, since all collected data from the focus groups and real‐world voice analysis were anonymized, there was no requirement for approval by an institutional review board.

## TRANSPARENCY STATEMENT

The lead author Margaret Yung affirms that this manuscript is an honest, accurate, and transparent account of the study being reported; that no important aspects of the study have been omitted; and that any discrepancies from the study as planned (and, if relevant, registered) have been explained.

## PREVIOUS PRESENTATIONS

The study has previously been presented at the 9th Congress of the European Academy of Neurology, held in Budapest, Hungary, July 1–4, 2023, and at the National Organization for Rare Diseases and Orphan Products Breakthrough Summit, held in Washington, DC, October 15–17, 2023.

## Supporting information

Supporting information.

Supporting information.

## Data Availability

Derived data supporting the findings are available from the corresponding author upon request.
