# Supplementary material for: Patient and care partner perspectives and preferences related to myasthenia gravis treatment: A qualitative study
Source: Health Sci Rep. 2024 Sep 24;7(9):e70081. doi: 10.1002/hsr2.70081 (PMC11422664; doi:10.1002/hsr2.70081)
Supplement: Supplementary file 1 — Supporting information. [file HSR2-7-e70081-s001.docx]

**Supporting Information 1. Pre-defined terms used in the real-world voice analysis**
