# Supplementary material for: Patient and care partner perspectives and preferences related to myasthenia gravis treatment: A qualitative study
Source: Health Sci Rep. 2024 Sep 24;7(9):e70081. doi: 10.1002/hsr2.70081 (PMC11422664; doi:10.1002/hsr2.70081)
Supplement: Supplementary file 2 — Supporting information. [file HSR2-7-e70081-s002.docx]

**Supporting Information 2. Questions used in pre-focus group questionnaire**

1. When you or your loved one are faced with a decision about a myasthenia gravis treatment, how important are these treatment attributes to you? Please rank in order from 1 (most important consideration) to 10 (least important consideration). [Rank order]
   1. How the treatment is given (self-injection, IV/infusion at medical center, infusion pump at home, etc.)
   2. Where the treatment is given (at home, at medical center, at hospital, etc.)
   3. How often the treatment needs to be given (once a day, once a week, once a month, once every two months, etc.)
   4. Impact on quality of life (emotional, social, and physical well-being)
   5. Controlling symptoms (daily changes, weekly changes, symptoms worsening)
   6. Avoiding or preventing a crisis (exacerbation)
   7. Expected length of time the treatment will work (a week, a month, etc.)
   8. How long it takes before improvement starts
   9. Reducing side effects from treatment
   10. Cost of treatment
2. When thinking about managing your or your loved one’s myasthenia gravis, please select the **top 5 factors** related to your/their current/most recent treatment that most concern you. [Select top five]
   1. Potential flare-ups/exacerbations/myasthenia gravis crises
   2. Mental health consequences associated with shift or change in symptoms
   3. Impact on quality of life (emotional, social, and physical well-being)
   4. Managing symptoms (impaired speech, chewing/swallowing, shortness of breath, general fatigue)
   5. How well the treatment will work
   6. How long it will take the treatment to start working
   7. Risk of side effects from treatment
   8. How is the treatment given (self-injection, IV/infusion at medical center, infusion pump at home, etc.)
   9. How often will the treatment need to be given
   10. Useful and clear treatment information shared by my provider
3. What are the treatment goals that are most important to you/your loved one. Please rank in order from 1 (most important) to 9 (least important) [Rank order]
   1. Reduced general fatigue
   2. Reduced mental fatigue/brain fog
   3. Improved short-term symptom stability (reducing shifts/changes in symptoms)
   4. Improve long-term symptom control
   5. Reducing impact of symptoms on daily life
   6. Reduced impact of symptoms on emotional/mental health
   7. Avoiding or preventing crisis (exacerbations)
   8. Returning to former activities (employment, hobbies, leisure, travel, etc.)
   9. Decrease use of steroid therapies (such as Prednisone) or other previous myasthenia gravis treatments
4. Do you stay up to date on **new or emerging** myasthenia gravis treatments?
   1. Yes
   2. No
   3. Sometimes
5. Please select the 5 most important expectations you have for **a new or emerging treatment** for myasthenia gravis. [Select top five]
   1. Reduced number and frequency of changing symptoms
   2. More predictability of myasthenia gravis symptoms
   3. Fewer myasthenia gravis crisis (exacerbations)
   4. Less side effects
   5. Medication you can use if you are pregnant or planning to become pregnant
   6. Being able to take the medication when it fits your schedule
   7. Being able to take the medication at home
   8. Less use of corticosteroids
   9. Feeling that the medication works quickly
   10. Having a medication that works in your body for a longer period of time
6. For **a new or emerging** myasthenia gravis treatment, how would you like it to be given to you/your loved one? Please rank each option in order from 1 (most preferred) to 5 (least preferred). [Likert scale 1-5, with pictures and explanations. Scale: 1 most preferred, 2 somewhat preferred, 3 neither preferred or not preferred, 4 somewhat not preferred, 5 least preferred)]

| **Please rank in order from 1 (most preferred) to 5 (least preferred)** | | |
| --- | --- | --- |
|  | 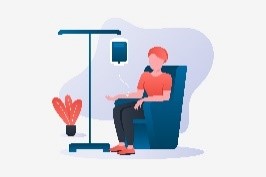 | Delivered IV in the hospital or an infusion center/clinic. |
|  | 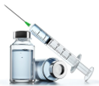 | Taken as a self-injection requiring preparation with a vial & syringe. |
|  | 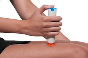 | Taken as a self-injection that is delivered subcutaneously (under the skin) via a preloaded device. |
|  | 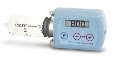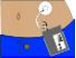 | Infusion pump where you insert a syringe into pump, attach the tubing and then inject into your body (requires preparation) |
|  | 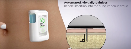 | On body delivery system that comes pre-assembled. You/your loved one will remove it from a box where the device will be left on your/their body for the medication to be given. The device will be disposed after a single use. |

1. How important are the following factors when making decisions about a next possible myasthenia gravis treatment for you/your loved one? [Likert scale question]

RATING SCALE / COLUMN HEADERS:

1. Not at all important
2. A little less important
3. Neither less or more important
4. A little more important
5. Very important

|  | **Factor** |
| --- | --- |
| 1 | Medication that is administered quickly (2 minutes vs 10-20 minutes) |
| 2 | Medication that requires a less frequent dosing schedule (every week vs every two weeks, etc.) |
| 3 | Medication delivery (needle you can see vs. one you cannot see) |
| 4 | Medication that starts working right away (1-2 weeks vs 1 month) |
| 5 | Medication device that works on its own (a digital read out controls the medication and tells you when it’s finished or when you can remove it) |
| 6 | Medication that does not require enrollment into a special monitoring program |
| 7 | Medication that does not require taking certain vaccines |

1. Which of the following statements best describes your preference today toward selecting treatments for myasthenia gravis? **Note:** For care partners, please answer Q8 from the perspective of your loved one.
   1. I prefer to make the final treatment selection about which treatment I receive
   2. I prefer to make the final selection of my treatment after seriously considering the doctor’s opinion
   3. I prefer that the doctor and I share responsibility for deciding which treatment is best for me
   4. I prefer that the doctor make the final decision about which treatment will be used but seriously consider my opinion
   5. I prefer to leave all decisions regarding my treatment to the doctor
